# The Anti-Caries Effects of a Novel Peptide on Dentine Caries: An In Vitro Study

**DOI:** 10.3390/ijms241814076

**Published:** 2023-09-14

**Authors:** Olivia Lili Zhang, John Yun Niu, Ollie Yiru Yu, Iris Xiaoxue Yin, May Lei Mei, Chun Hung Chu

**Affiliations:** 1 Faculty of Dentistry, The University of Hong Kong, Hong Kong 999077, China; zhlili@connect.hku.hk (O.L.Z.); niuyun@hku.hk (J.Y.N.); ollieyu@hku.hk (O.Y.Y.); irisxyin@hku.hk (I.X.Y.); may.mei@otago.ac.nz (M.L.M.); 2 Faculty of Dentistry, The University of Otago, Dunedin 9054, New Zealand

**Keywords:** antimicrobial, caries, demineralization, peptides, prevention, remineralization

## Abstract

This study aimed to investigate the antibiofilm and remineralising effects of peptide GAPI on artificial dentin caries. After creating artificial carious lesions, eighty dentine blocks were randomly assigned for treatment twice daily with GAPI (GAPI group) or deionised water (control group). Both groups underwent a 7-day biochemical cycle. Scanning electron microscopy (SEM) showed *S. mutans* with damaged structures that partially covered the dentine in the GAPI group. The dead–live ratios for the GAPI and control groups were 0.77 ± 0.13 and 0.37 ± 0.09 (*p* < 0.001). The log colony-forming units for the GAPI and control groups were 7.45 ± 0.32 and 8.74 ± 0.50 (*p* < 0.001), respectively. The lesion depths for the GAPI and control groups were 151 ± 18 µm and 214 ± 15 µm (*p* < 0.001), respectively. The mineral losses for the GAPI and control groups were 0.91 ± 0.07 gHAcm^−3^ and 1.01 ± 0.07 gHAcm^−3^ (*p* = 0.01), respectively. The hydrogen-to-amide I ratios for the GAPI and control groups were 2.92 ± 0.82 and 1.83 ± 0.73 (*p* = 0.014), respectively. SEM micrographs revealed fewer exposed dentine collagen fibres in the GAPI group compared to those in the control group. Furthermore, X-ray diffraction (XRD) patterns indicated that the hydroxyapatite in the GAPI group was more crystallised than that in the control group. This study demonstrated GAPI’s antibiofilm and remineralising effects on artificial dentin caries.

## 1. Introduction

Caries is a non-communicable dental disease that results from the dysbiosis of the resident oral microbiota [1,2]. Aciduric and acidogenic species, such as *Streptococcus mutans*, are essential for cariogenic bacteria colonisation to develop cariogenic biofilms [3]. *S. mutans* can rapidly adhere to the teeth and assemble a biofilm by producing insoluble extracellular polysaccharides [4]. In addition, *S. mutans* produce acids by consuming sugar and reducing the local pH to below 5.5, leading to the demineralisation of the mineral [5]. Normally, the saliva buffer system can adjust the balance between remineralisation and demineralisation. However, caries progresses if this balance is disrupted due to the preponderance of pathological factors [6]. Therefore, it is essential to control a cariogenic biofilm to manage dental caries. Moreover, preventing the demineralisation of the tooth’s hard tissue and promoting the remineralisation of the demineralised tooth hard tissue is important.

The philosophy of caries management has developed into minimal intervention, which means prolonging the tooth’s life by maintaining its structure and pulpal vitality [7]. Therefore, scientists are exploring the development of innovative bioactive materials for the management of dental caries [8]. Antimicrobial peptides are one of the bioactive materials that could be considered to be a favourable strategy for caries management [9]. Various species, such as animals, plants, and microorganisms, produce antimicrobial peptides as a defence mechanism against pathogens [10]. These peptides directly bind to microbial surfaces and display broad-spectrum activity against pathogens [11]. Moreover, after penetrating the cytoplasm, antimicrobial peptides can interact with intracellular substances, inhibiting intracellular enzyme activity and disrupting nucleic acid, proteins, and cell wall synthesis [12,13]. Additionally, antimicrobial peptides are less likely to generate resistance due to their ability to attack multiple hydrophobic and polyanionic bacterial targets [14,15].

Several in vitro studies have shown natural antimicrobial peptides’ efficiency in killing cariogenic bacteria [16]. One potential approach to enhancing the application of antimicrobial peptides for managing caries is to create new peptides that mimic natural antimicrobial peptides [9]. For example, Zhang et al. developed a new tooth-binding peptide by incorporating phosphoserine residues into the peptide polyphemusin I [17]. This peptide can bind to the tooth surface to endorse prolonged antimicrobial activities. Another example is Guo et al., who synthesised C16G2 using an *S. mutans*-targeting peptide (C16) and broad-spectrum antimicrobial peptide (G2) [18]. This peptide can specifically target *S. mutans* [19]. However, these novel peptides lack mineralising properties. Thus, developing novel dual-action peptides with antimicrobial and mineralising properties is an expected strategy for developing novel peptides for caries management.

In a previous study, we developed a novel and biocompatible peptide called GAPI, which possesses antimicrobial and mineralising abilities. We created the GAPI peptide by attaching gallic acid as the remineralising domain to the antimicrobial peptide polyphemusin I. Polyphemusin I is an antimicrobial peptide which can kill bacteria and fungi. It is derived from horseshoe crabs. As a β-sheet peptide, Polyphemusin I can bind to and cross cell membranes, thus rupturing the microbial membrane [17]. Gallic acid is abundant in fruits and vegetables. It can accelerate the regeneration of hydroxyapatites due to its pyrogallol group and has antimicrobial activities [14]. The GAPI peptide can be synthesised using the standard fluorenylmethoxycarbonyl solid-phase synthesis method. The purity of synthesised GAPI could reach 96.74% and its molecule weight was 2608.05 kD. The secondary structure analysis of GAPI showed that it is a β-sheet peptide. Thus, it should be an effective antimicrobial peptide. Our study demonstrated that GAPI can inhibit the growth of common oral pathogens and the formation of cariogenic biofilms under laboratory conditions [20]. Moreover, GAPI enhanced the remineralisation of demineralised enamel in a chemical model. However, the progression of caries on enamel or dentin differs.

Dentin, unlike enamel, is a calcified tissue with an organic matrix consisting of 90% collagen and 10% non-collagenous proteins [21]. During the progress of dentine caries, the mineral component dissolves, whereas the organic matrix deteriorates [22]. GAPI’s effectiveness on dentin caries remains uncertain. Therefore, this study’s objective was to assess GAPI’s antibiofilm and remineralising effects on artificial dentin caries using a biofilm remineralisation cycling model involving *S. mutans* [14]. For this study, we used the *S. mutans* mono-species biofilm challenge to create the initial dentine caries and then incorporated it into the *S. mutans* biofilm remineralisation cycling model. Although a mono-species biofilm cannot fully replicate real-life scenarios, this study was an initial laboratory investigation.

## 2. Results

A total of eighty dentine blocks were prepared for this study. After a 3-day *S.mutans* biofilm challenge at 37 °C, an artificial caries lesion surface, with around a 96 μm depth, was successfully created. Then, all the dentine blocks were allocated into the GAPI-treated group (160 μM GAPI) and the control group (deionised water). All the blocks underwent a biochemical model involving chemical remineralisation and a *S. mutans* biofilm challenge for seven days.

### 2.1. Effect of GAPI Peptide on Biofilm

**Figure 1:**
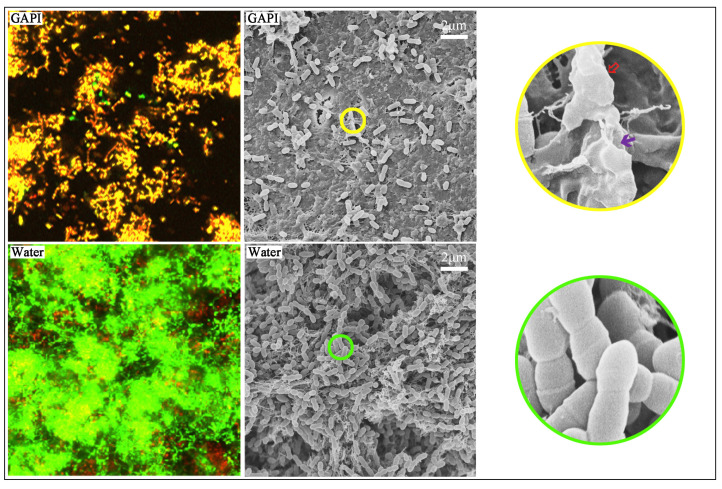
CLSM images (×100) and SEM images (×2000 and ×20,000) of the *S. mutans* biofilm. The CLSM images showed dead (red)/live (green) fluorescence of the *S. mutans* biofilm. The dead–live ratio for the GAPI-treated group was significantly higher than that for the control group (*p* < 0.001). The SEM images showed the structure of the *S. mutans* biofilm. The bacterial coverage in the GAPI-treated group was lower than that in the control group. In addition, GAPI treatment disrupted the bacterial structure, including loss of normal bacterial cell morphology (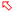) and damage to bacterial cell membranes (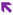).

The confocal laser scanning microscopy (CLSM) images displayed an increased red fluorescence of *S. mutans* on the dentin in the GAPI-treated group. On the other hand, the CLSM images exhibited almost entirely green fluorescence of *S. mutans* on the dentin in the control group (Figure 1). 

When the dead–live ratios from the CLSM images were computed, the ratios for the GAPI and control groups were 0.77 ± 0.13 and 0.37 ± 0.09, respectively (*p* < 0.001). These findings suggested that GAPI significantly hindered the growth of *S. mutans* on the dentin surface. Table 1 shows the log CFUs representing the growth kinetics of *S. mutans* in both groups. The log CFUs were 7.45 ± 0.32 and 8.74 ± 0.50 (*p* < 0.001) for the GAPI and control groups, respectively. 

**Figure 2:**
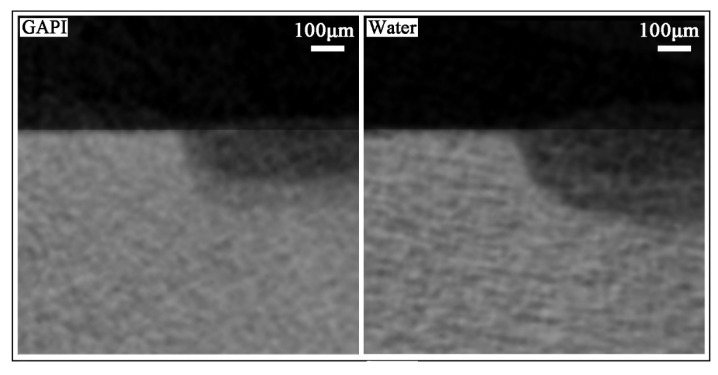
Micro-CT images of dentine lesion treated with GAPI (**Left**) and water (**Right**). The lesion depth for the GAPI-treated group was significantly lower than that in the control group (*p* < 0.001).

In the low-magnification scanning electron microscopy (SEM) images, the bacterial coverage on the dentin surface in the GAPI group was lower than that in the control group (Figure 2). In the control group, a three-dimensional *S. mutans* biofilm entirely covered the dentin surface, with the bacterial cells being tightly connected to each other. In the high-magnification SEM images, the GAPI treatment disrupted the bacterial structure, including a loss of normal bacterial cell morphology and damage to the bacterial cell membranes. Conversely, the bacterial cells remained intact in the control group.

### 2.2. Effects of GAPI Peptide on Hard Tissue

Micro-computed tomography (micro-CT), Fourier transform infrared (FTIR), SEM, and X-ray diffraction (XRD) were used to assess the lesion depths, mineral losses, changes in chemical structure, dentine surfaces and cross-section morphologies, and crystal characteristics on the dentine blocks’ carious lesions, respectively. Table 1 presents the average lesion depth and average mineral loss. The lesion depths for the GAPI and control groups were 151 ± 18 µm and 214 ± 15 µm, respectively (*p* < 0.001). The mineral losses for the GAPI and control groups were 0.91 ± 0.07 gHAcm^−3^ and 1.01 ± 0.07 gHAcm^−3^, respectively (*p* = 0.01). Figure 2 shows the representative micro-CT images. The dentin blocks treated with GAPI exhibited a notably lower average lesion depth and mineral loss compared to those of the control group.

**Figure 3:**
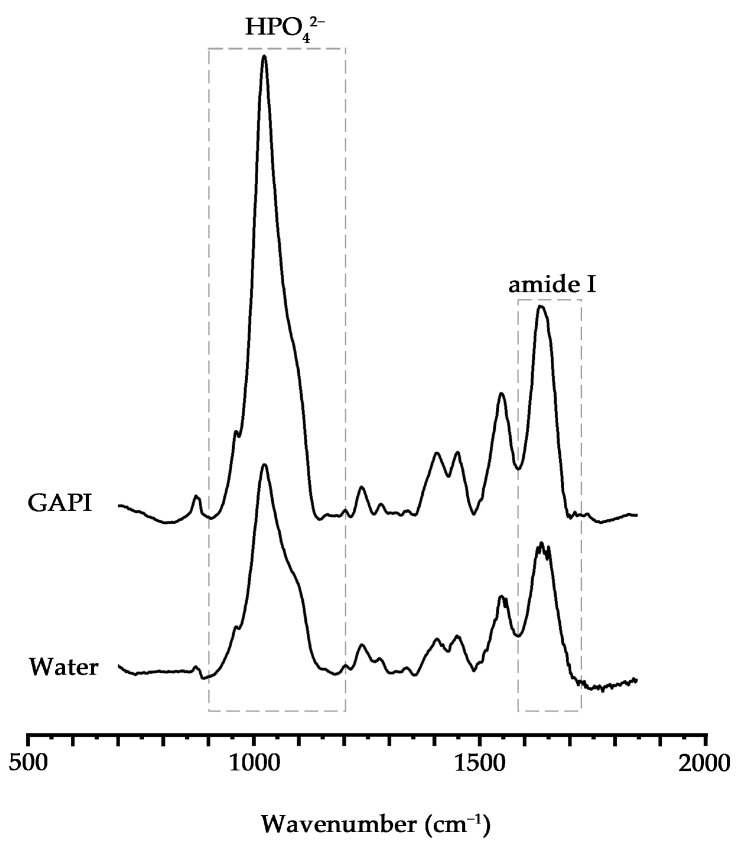
Fourier transform infrared spectra of the dentine caries lesion. The HPO_4_^2−^ band wavelength from 900 to 1200 cm ^−1^ and the amide I band wavelength from 1585 to 1720 cm ^−1^. The HPO_4_^2 −^-to-amide I ratio for the GAPI-treated group was significantly higher than that of the control group (*p* = 0.014).

The average HPO_4_^2−^-to-amide I ratios for the GAPI and control groups were 2.92 ± 0.82 and 1.83 ± 0.73 (*p* = 0.014). This indicates that the dentin in the control group experienced a greater mineral tissue loss than that in the GAPI group. Figure 3 displays the characteristic Fourier transform infrared spectroscopy (FTIR) spectra of both groups.

**Figure 4:**
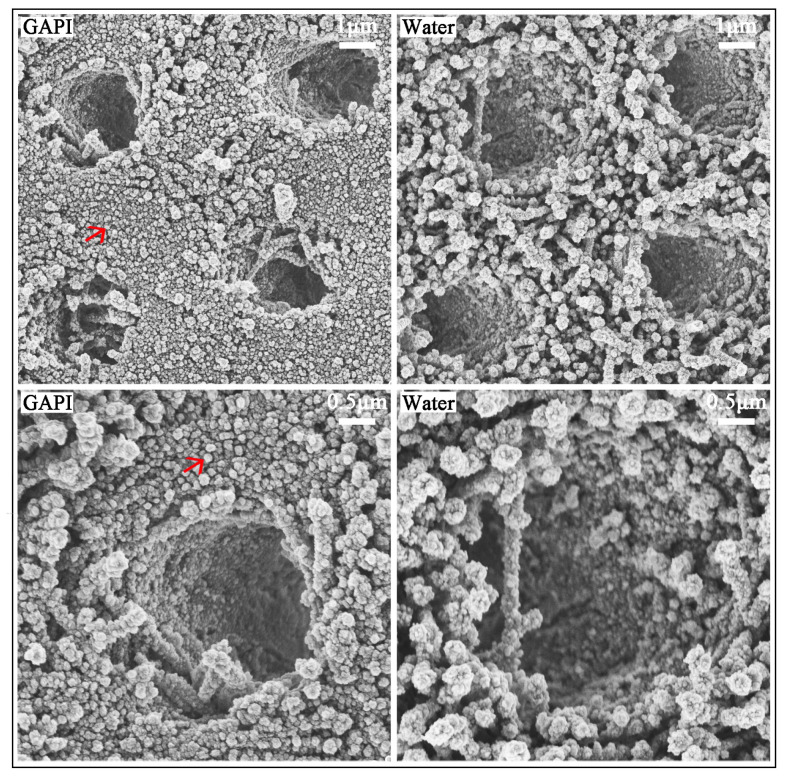
SEM images (×7000 and ×15,000) of dentine treated with GAPI (**Left**) and water (**Right**). In the GAPI group, the dentin surface was relatively smooth with the ball-form mineral nodes (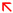) indicating remineralisation. In contrast, the dentin surface appeared rough with serious exposure of collagen fibres in the control group.

Figure 4 displays the typical dentin surface morphology. In the GAPI group, the dentin surface was relatively smooth, with slight collagen fibre exposure. In addition, the ball-form mineral nodes on the dentine in the GAPI group indicated remineralisation. In contrast, the dentin surface appeared to be rough, and the exposure of collagen fibres was significantly evident in the control group.

**Figure 5:**
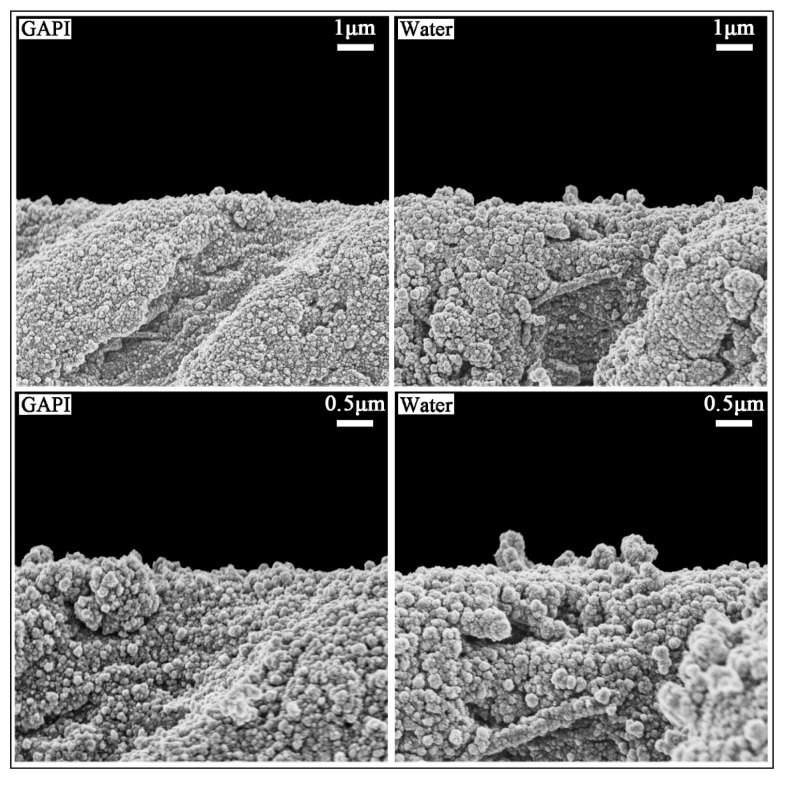
SEM images (×7000 and ×15,000) of cross-section dentine treated with GAPI (**Left**) and water (**Right**). The dentinal tubular and inter-tubular regions were relatively full of mineral nodes in the GAPI-treated group. In contrast, the dentin collagen fibre was exposed in the control group.

Figure 5 shows typical dentine’s cross-sectional morphology. The cross-sectional images exhibited that the dentinal tubular and inter-tubular regions were relatively full of mineral nodes, with slight dentin collagen fibre exposure in the GAPI group. 

**Figure 6:**
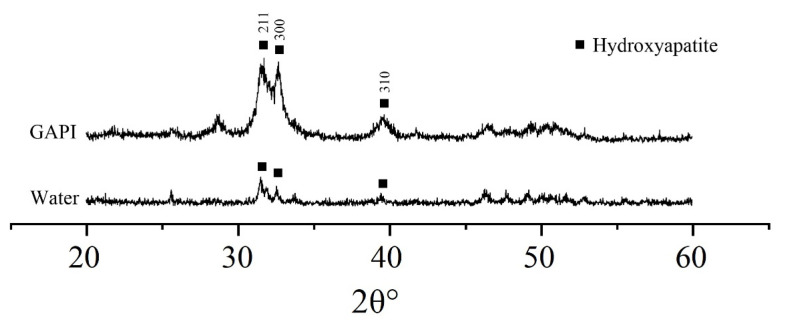
X-ray diffraction patterns of the dentine caries lesion. The hydroxyapatite diffraction peaks including 211, 300, and 310 were identified in the GAPI-treated group, suggesting the existence of hydroxyapatite crystals. In contrast, the control group showed no prominent diffraction peaks.

Figure 6 displays the typical characteristic XRD spectra of dentin. In the GAPI group, hydroxyapatite diffraction peaks (211, 300, and 310) were identified at 31.8°, 32.9°, and 39.8°. These peaks suggested the existence of hydroxyapatite crystals. In contrast, the spectra in the control group showed no prominent diffraction peaks.

## 3. Discussion

Recently, antimicrobial peptides have been regarded as a bioactive material for developing novel anti-caries agents [23]. Polyphemusin I is a broad-spectrum antimicrobial peptide used against *S. mutans* [17]. It could be an ideal template peptide for developing novel anti-caries peptides. Gallic acid with a pyrogallol group can attract calcium from the microenvironment to enhance hydroxyapatite regeneration. In a previous study, we developed and synthesised a novel peptide, GAPI, by attaching gallic acid to polyphemusin I. It is essential to investigate GAPI’s impact on dentine caries, considering its antibiofilm and remineralising effects. We showed that GAPI possesses antimicrobial and mineralising properties and is capable of inhibiting the growth of cariogenic biofilms and promoting the remineralisation of initial enamel caries in a chemical model. However, chemical models, such as pH-cycling models, lack biological factors. Thus, we utilised a biofilm remineralisation cycling model involving *S. mutans* in this study. This unique model incorporated both biological and chemical factors to create pH changes and establish a microbiological environment that mimicked bacterial impacts in the oral cavity. The effectiveness of this model in simulating the oral environment was demonstrated. The *S. mutans* biofilm is a frequently employed microbial model for caries research. In this study, we utilised an *S. mutans* mono-species biofilm challenge to induce demineralisation and create initial dentine caries, which was then incorporated into the *S. mutans* biofilm remineralisation cycling model. In order to produce acid, 5% sucrose was added to the bacterial culture. Furthermore, to mimic a high-risk caries scenario, we subjected the dentine to a 16 h demineralisation process. 

GAPI’s antibiofilm effect on the *S. mutans* biofilm on the dentin surface was evaluated by assessing the viability, growth kinetics, and morphology. A quantitative analysis of the dead–live ratio and log CFUs revealed a significant difference between the two groups. These findings suggested that GAPI could effectively inhibit the growth of the *S. mutans* biofilm on the dentin. The SEM images of the biofilm further confirmed that GAPI could damage the bacterial cell membrane, resulting in the loss of a typical bacterial structure and the leakage of cytoplasmic content. This mechanism was related to the antimicrobial function domain, polyphemusin I, which is derived from horseshoe crabs with a positive net charge [24]. Amiss et al. indicated that polyphemusin I can effectively kill bacteria with lower concentrations in extracellular and intracellular environments [25]. Polyphemusin I is known as one of the cell-penetrating peptides due to its β-hairpin structure. Cell-penetrating peptides typically consist of 5–30 amino acids, allowing them to cross cell membranes and interact with intracellular targets [26]. The results of this study aligned with our previous research, suggesting that the GAPI peptide has potential as an antimicrobial agent for caries management.

GAPI’s mineralising effects on dentin caries manifested in promoting remineralisation, impeding demineralisation, and preventing collagen degradation. These effects could be examined through lesion depth, mineral loss, chemical structure, crystal properties, and surface morphology. Cross-sectional morphology can help to explain collagen degradation. In this study, micro-CT, a non-invasive testing technique, was used to assess the dentin’s mineral content. The micro-CT results, such as those for lesion depth and mineral loss, demonstrated less demineralisation in the GAPI group. Moreover, we used FTIR to analyse the dentin blocks’ chemical structures. In the FTIR spectra, the amide I band represented collagen and the phosphate band signified the mineral matrix. The HPO_4_^2−^-to-amide I ratio was correlated with the degree of demineralisation, with higher ratios indicating reduced demineralisation. In this study, the HPO_4_^2−^-to-amide I ratio in the GAPI group was significantly higher than that in the control group. In addition, the amide I band area in the GAPI group exceeded than that in the control group. These findings indicated that GAPI effectively inhibited demineralisation and collagen degradation. The observed effect of impeding demineralisation and preventing collagen degradation can be partly ascribed to the lower bacterial burden given by the antimicrobial activity of the peptide. Moreover, the SEM results revealed the presence of mineral nodes in the GAPI group, confirming that GAPI promoted the formation of extra-fibrillar minerals.

Due to dentin’s unique composition, the collagen structure plays a crucial role as a scaffold for the deposition of mineral crystals. Mineral crystals and calcium resources are also essential. Therefore, these factors need to be considered in dentin caries treatment. Fluoride-based materials, such as silver diamine fluoride, are commonly applied in caries management and can be incorporated into fluorapatite crystals, enhancing the remineralising process [8]. However, this may lead to black discoloration on the tooth [8]. Other bioactive substances, such as calcium- and phosphate-based materials, including casein phosphopeptide-amorphous calcium phosphate and nano-hydroxyapatite, act as sources of calcium and phosphate for remineralising the demineralised dentin surface [27]. GAPI can suppress the growth of cariogenic biofilms, thereby reducing acid production. This mechanism can hinder the dissolution of hydroxyapatite and prevent collagen degradation. In addition, the pyrogallol group of GAPI can help to attract calcium and phosphate to promote remineralisation. Overall, the fusing peptide GAPI offers a promising strategy for anti-caries management. Additional investigations and clinical trials are necessary to further evaluate the application of GAPI for dentin caries.

## 4. Materials and Methods

### 4.1. Synthesisation of Peptide

GAPI was synthesised using standard fluorenylmethoxycarbonyl synthesis through conventional solid-phase peptide synthesis. The GAPI powder was dissolved in sterile deionised water and stored at −20 °C.

### 4.2. Preparation of Dentine Blocks with Artificial Carious Lesions

Dentine slices, 2 mm thick, were cut from sound extracted third molars. Ultra-fine 4000-grit sandpaper was used to polish these slices. Slices with defects were eliminated based on a stereomicroscope observation. A total of forty dentine slices were selected. Each slice was separated into two blocks and assigned to two groups. Nail varnish was used to coat half of the blocks as an internal control [14]. *S. mutans* American Type Culture Collection 35668 was utilised in the present study. All the dentine blocks were placed into *S. mutans* culture (10^8^ cells/mL) in a brain–heart infusion (BHI) broth containing 5% glucose to generate artificial caries. After a 3-day anaerobic culture at 37 °C, the depth of the caries lesion surface was around 96 μm, which was observed using micro-computed tomography (micro-CT).

### 4.3. Experimental Treatment

**Figure 7:**
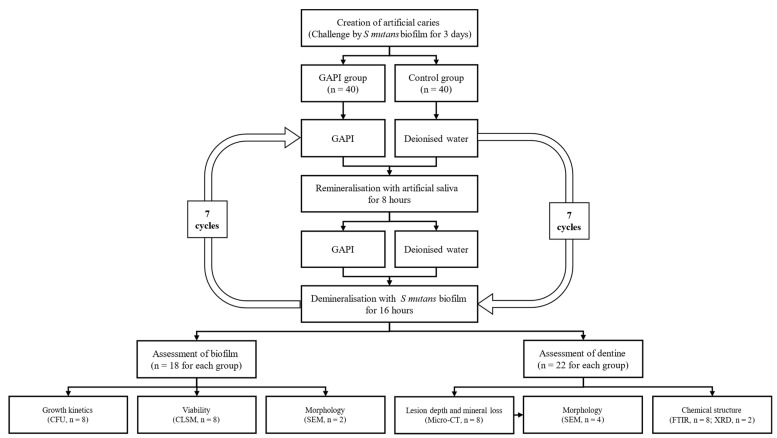
Study protocol.

Two blocks from the same slice were assigned to the GAPI group (160 μM GAPI) and control group (deionised water) separately. All the blocks underwent a biochemical model involving chemical remineralisation and an *S. mutans* biofilm challenge for seven days. The remineralisation solution (pH 7.0) consisted of 1.5 mm calcium chloride, 150 mm potassium chloride, 0.9 mm potassium dihydrogen phosphate, and 20 mm 4-(2-hydroxyethyl)-1-piperazineethanesulfonic acid. The cycling process included 8 h of immersion in the remineralisation solution and 16 h of immersion in an *S. mutans* biofilm medium. Figure 7 shows the research protocol of this study.

Before the solutions were transformed, each block was washed in an ultrasonic bath. The blocks in the GAPI group received a 5 min topical application of GAPI using micro brushes (ROCODENT, Foshan, China), and the blocks in the control group received deionised water [14]. 

### 4.4. Antibiofilm Effect of GAPI Peptide

#### 4.4.1. Biofilm Viability

CLSM (Fluoview FV 1000, Olympus, Tokyo, Japan) was utilised to capture images to assess the *S. mutans* biofilm viability (*n* = 8 for each group). Two fluorescent probes were used to label the bacteria (LIVE/DEAD BacLight Bacterial viability kit, Molecular Probes, Eugene, OR, USA). Among these probes, the propidium iodide probe stained the dead bacterial cells red, and the SYTO-9 probe stained the live bacterial cells green. The red–green ratio represented the dead–live ratio calculated using Image J version 1.53t (National Institutes of Health, Bethesda, MD, USA) [14].

#### 4.4.2. Biofilm Kinetics

To determine the *S. mutans* biofilm growth kinetics, the colony-forming units (CFUs) were counted (*n* = 8 for each group). The dentin blocks that underwent biochemical cycles were slightly rinsed with a phosphate-buffered solution. Then, the blocks were shaken in a vortex machine to collect the bacterial cells in a 1 mL BHI broth. Subsequently, a ten-fold serial dilution of the bacterial solution was prepared, and 10 μL of each dilution was plated on blood agar. All the plates were cultured anaerobically for 48 h at 37 °C before the CFUs were counted. The log CFU was calculated for a statistical analysis.

#### 4.4.3. Biofilm Morphology

SEM (Hitachi S-4800 FEG Scanning Electron Microscope; Hitachi, Tokyo, Japan) was employed to evaluate the morphology of the *S. mutans* biofilm (*n* = 2 for each group). Each dentin block was fixed in a 2.5% glutaraldehyde solution for 4 h at 4 °C. Following dehydration, all the blocks were critical point dried in a desiccator and coated using a sputter coater [14].

### 4.5. Effect of GAPI Peptide on Hard Tissue

#### 4.5.1. Lesion Depth and Mineral Loss

Micro-CT (SkyScan 1272, Antwerp, Belgium) was employed to evaluate the lesion depths and mineral losses (*n* = 8 for each group). The X-ray source parameters were set at an 80 kV voltage and 100 μA current with an image pixel size of 10 μm. Moreover, two standard mineral cylindrical phantoms (Bruker, Kontich, Belgium) with mineral density values (MDVs, gHApcm^−3^) of 0.25 gHApcm^−3^ and 0.75 gHApcm^−3^ were scanned to calibrate the blocks’ greyscales. The NRecon reconstruction software (Version 1.7.4.6, SkyScan, Antwerp, Belgium) was utilised to reconstruct the scanning images. The CTAn data analysis software (CTAn version 1.20.3.0, Skyscan NV, Kontich, Belgium) was employed to analyse the cross-sectional images. The lesion depths were measured, and the images’ greyscale values were calibrated. Subsequently, the demineralised and control areas were calculated into the MDV for each block. The mineral loss was determined as the difference between the MDV of the control and the MDV of the demineralised area [14].

#### 4.5.2. Chemical Structure

FTIR (Spectrum Two, PerkinElmer, Waltham, MA, USA) was employed to examine the changes in the dentin’s chemical structures (*n* = 8 for each group). The infrared radiation wavelengths ranged from 550 to 2000 cm^−1^, the HPO4^2−^ band wavelength from 900 to 1200 cm^−1^, and the amide I band wavelength from 1585 to 1720 cm^−1^. The ratio of the HPO4^2−^ to amide I absorbance area represented the degree of demineralisation in the dentin.

#### 4.5.3. Surface Morphology and Cross-Sectional Morphology

SEM (Hitachi S-4800 FEG Scanning Electron Microscope; Hitachi, Tokyo, Japan) was employed to evaluate the dentin surface morphology (*n* = 2 for each group) and cross-sectional topography (*n* = 2 for each group). Four blocks from each group were ultrasonically cleaned in distilled water to remove the biofilm prior to its fixation in 2.5% glutaraldehyde at 4 °C for 4 h. Of the four blocks, two were fractured in half in liquid nitrogen for an observation of their cross-sectional topography. A series of ethanol solutions was used to dehydrate the blocks. Subsequently, the blocks were dried in a desiccator and coated with carbon using a sputter coater [14].

#### 4.5.4. Crystal Characteristics

X-ray diffraction (XRD, Rigaku SmartLab 9 kW with CuKa [l = 1.5418 Å], Bruker AXS GmbH, Karlsruhe, Germany) was employed to examine the dentin’s crystal properties. Scans were conducted with a range of 20–60° 2θ and a step size of 0.05° at a scanning speed of 30 s/step. The phase purity and indexing of the chemical phase were verified using the International Centre for Diffraction Data database (PDF-2 Release 2004) [14].

### 4.6. Statistical Analysis

All the data were digitised and analysed using the SPSS version 23 (IBM Corp, Armonk, NY, USA). The Shapiro–Wilk test was conducted to assess the normality of the distribution. A two-sample *t*-test was employed to measure the differences in the dead-to-live ratios, log CFUs, lesion depths, mineral losses, and HPO4^2−^ to amide I ratios between the two groups. The significance level was set at 5%.

## 5. Conclusions

This in vitro study showed that the peptide GAPI is capable of hindering *S. mutans* biofilm development on dentine surfaces. In addition, GAPI promoted remineralisation, impeded demineralisation, and prevented the collagen degradation of the dentine. All of these effects demonstrated that GAPI can prevent the demineralisation and enhance the remineralisation of artificial caries on dentin. The promising effects of GAPI demonstrated its potential to act as an anti-caries agent for clinical use.

## Figures and Tables

**Table 1:** *S. mutans* biofilm and carious dentine lesions treated with GAPI and water.

Group	GAPI	Water	*p* Value
*S. mutans biofilm assessment*			
Viability: Dead-to-live ratio (*n* = 8)	0.77 ± 0.13	0.37 ± 0.09	<0.001
Kinetics: Log_10_ CFU (*n* = 8)	7.45 ± 0.32	8.74 ± 0.50	<0.001
*Carious dentine lesion assessment*			
Lesion depth, µm (*n* = 8)	151 ± 18	214 ± 15	<0.001
Mineral loss, gHAcm^−3^ (*n* = 8)	0.91 ± 0.07	1.01 ± 0.07	0.01
Hydrogen phosphate-to-amide I ratio (*n* = 8)	2.92 ± 0.82	1.83 ± 0.73	0.014

## Data Availability

Data are contained within the article.

## References

[1] Caufield P.W., Li Y., Dasanayake A. (2005). Dental caries: An infectious and transmissible disease. Compend. Contin. Educ. Dent..

[2] Machiulskiene V., Campus G., Carvalho J.C., Dige I., Ekstrand K.R., Jablonski-Momeni A., Maltz M., Manton D.J., Martignon S., Martinez-Mier E.A. (2020). Terminology of Dental Caries and Dental Caries Management: Consensus Report of a Workshop Organized by ORCA and Cariology Research Group of IADR. Caries Res..

[3] Chen X., Daliri E.B.-M., Tyagi A., Oh D.-H. (2021). Cariogenic Biofilm: Pathology-Related Phenotypes and Targeted Therapy. Microorganisms.

[4] Koo H., Xiao J., Klein M.I., Jeon J.G. (2010). Exopolysaccharides Produced by Streptococcus mutans Glucosyltransferases Modulate the Establishment of Microcolonies within Multispecies Biofilms. J. Bacteriol..

[5] Shellis R.P., Barbour M.E., Jones S.B., Addy M. (2010). Effects of pH and acid concentration on erosive dissolution of enamel, dentine, and compressed hydroxyapatite. Eur. J. Oral Sci..

[6] Takahashi N., Nyvad B. (2011). The Role of Bacteria in the Caries Process: Ecological perspectives. J. Dent. Res..

[7] Federation F.W.D. (2017). FDI policy statement on Minimal Intervention Dentistry (MID) for managing dental caries: Adopted by the General Assembly: September 2016, Poznan, Poland. Int. Dent. J..

[8] Zhang O.L., Niu J.Y., Yin I.X., Yu O.Y., Mei M.L., Chu C.H. (2023). Bioactive Materials for Caries Management: A Literature Review. Dent. J..

[9] Zhang O.L., Niu J.Y., Yin I.X., Yu O.Y., Mei M.L., Chu C.H. (2022). Growing Global Research Interest in Antimicrobial Peptides for Caries Management: A Bibliometric Analysis. J. Funct. Biomater..

[10] Kumar P., Kizhakkedathu J.N., Straus S.K. (2018). Antimicrobial Peptides: Diversity, Mechanism of Action and Strategies to Improve the Activity and Biocompatibility In Vivo. Biomolecules.

[11] Lei J., Sun L., Huang S., Zhu C., Li P., He J., Mackey V., Coy D.H., He Q. (2019). The antimicrobial peptides and their potential clinical applications. Am. J. Transl. Res..

[12] Mai S., Mauger M.T., Niu L.-N., Barnes J.B., Kao S., Bergeron B.E., Ling J.-Q., Tay F.R. (2017). Potential applications of antimicrobial peptides and their mimics in combating caries and pulpal infections. Acta Biomater..

[13] Zhang Q.-Y., Yan Z.-B., Meng Y.-M., Hong X.-Y., Shao G., Ma J.-J., Cheng X.-R., Liu J., Kang J., Fu C.-Y. (2021). Antimicrobial peptides: Mechanism of action, activity and clinical potential. Mil. Med. Res..

[14] Niu J.Y., Yin I.X., Wu W.K.K., Li Q.-L., Mei M.L., Chu C.H. (2022). Efficacy of the dual-action GA-KR12 peptide for remineralising initial enamel caries: An in vitro study. Clin. Oral Investig..

[15] Pfalzgraff A., Brandenburg K., Weindl G. (2018). Antimicrobial Peptides and Their Therapeutic Potential for Bacterial Skin Infections and Wounds. Front. Pharmacol..

[16] Wang Z., Shen Y., Haapasalo M. (2017). Antibiofilm peptides against oral biofilms. J. Oral Microbiol..

[17] Zhang L.-Y., Fang Z.-H., Li Q.-L., Cao C.Y. (2019). A tooth-binding antimicrobial peptide to prevent the formation of dental biofilm. J. Mater. Sci. Mater. Med..

[18] Guo L., McLean J.S., Yang Y., Eckert R., Kaplan C.W., Kyme P., Sheikh O., Varnum B., Lux R., Shi W. (2015). Precision-guided antimicrobial peptide as a targeted modulator of human microbial ecology. Proc. Natl. Acad. Sci. USA.

[19] Namburu J.R., Sanosh A.B.R., Poosarla C.S., Manthapuri S., Pinnaka M., Baddam V.R.R. (2022). Streptococcus mutans-Specific Antimicrobial Peptide C16G2-Mediated Caries Prevention: A Review. Front. Dent..

[20] Zhang O.L., Niu J.Y., Yin I.X., Yu O.Y., Mei M.L., Chu C.H. (2023). Antibacterial Properties of the Antimicrobial Peptide Gallic Acid-Polyphemusin I (GAPI). Antibiotics.

[21] Goldberg M., Kulkarni A.B., Young M., Boskey A. (2011). Dentin structure composition and mineralization. Front. Biosci..

[22] Mazzoni A., Tjäderhane L., Checchi V., Di Lenarda R., Salo T., Tay F., Pashley D., Breschi L. (2014). Role of Dentin MMPs in Caries Progression and Bond Stability. J. Dent. Res..

[23] Raheem N., Straus S.K. (2019). Mechanisms of Action for Antimicrobial Peptides with Antibacterial and Antibiofilm Functions. Front. Microbiol..

[24] Edwards I.A., Elliott A.G., Kavanagh A.M., Zuegg J., Blaskovich M.A.T., Cooper M.A. (2016). Contribution of Amphipathicity and Hydrophobicity to the Antimicrobial Activity and Cytotoxicity of β-Hairpin Peptides. ACS Infect. Dis..

[25] Amiss A.S., von Pein J.B., Webb J.R., Condon N.D., Harvey P.J., Phan M.-D., Schembri M.A., Currie B.J., Sweet M.J., Craik D.J. (2021). Modified horseshoe crab peptides target and kill bacteria inside host cells. Cell. Mol. Life Sci..

[26] Guidotti G., Brambilla L., Rossi D. (2017). Cell-Penetrating Peptides: From Basic Research to Clinics. Trends Pharmacol. Sci..

[27] González-Cabezas C., Fernández C. (2018). Recent Advances in Remineralization Therapies for Caries Lesions. Adv. Dent. Res..

